# ERRATUM

**DOI:** 10.1111/os.12829

**Published:** 2020-11-13

In [1], the first affiliation was erroneously published as “Department of Orthopaedics, People's Hospital of Guizhou Province, Guiyang, Guangzhou, China”.

The correct affiliation should be “Department of Orthopaedics, People's Hospital of Guizhou Province, Guiyang, Guizhou, China.”

The author apologizes for the error and any convenience it may have caused.
